# Role of MMP-2, MMP-9, TIMP-1, and TIMP-2 in children with ventricular septal defect

**DOI:** 10.17305/bb.2024.11162

**Published:** 2024-10-17

**Authors:** Nina Maric, Ines Mrakovcic Sutic, Jelica Predojevic Samardzic, Dario Djukic, Aleksandar Bulog, Ivana Sutic

**Affiliations:** 1 Clinic for Children’s Diseases, University Clinical Centre of the Republic of Srpska, Banja Luka, Bosnia and Herzegovina; 2 Department of Paediatrics, Medical Faculty, University of Banja Luka, Banja Luka, Bosnia and Herzegovina; 3 Departmant of Physiology, Immunology and Pathophysiology, Faculty of Medicine, University of Rijeka, Rijeka, Croatia; 4 Department of Health Ecology, Faculty of Medicine, University of Rijeka, Rijeka, Croatia; 5 Institute of Public Health, Primorsko-Goranska County, Rijeka, Croatia; 6 Department of Family Medicine, Medical Faculty, University of Rijeka, Rijeka, Croatia

**Keywords:** Ventricular septal defect (VSD), matrix metalloproteinases (MMPs), tissue inhibitor of metalloproteinases, urine

## Abstract

Ventricular septal defect (VSD) is the second most common congenital heart anomaly. In most cases, it closes spontaneously in the first year of life, but it sometimes requires surgical closure due to the risk of serious complications. This is why it is important to identify markers that could help predict its course. Findings that matrix metalloproteinases (MMPs) and tissue inhibitors of metalloproteinases (TIMPs) play an important role in the cleavage of the extracellular matrix were the reasons to investigate their role in cardiogenesis. In prior studies on this topic, their concentrations were studied in the blood. The aim of this prospective study was to investigate the role of MMP-2, MMP-9, TIMP-1, and TIMP-2 in the etiology and pathophysiology of VSD using urine samples, as an innovative non-invasive approach, and the enzyme-linked immunosorbent assay (ELISA) method. It involved 52 children with isolated VSD and 20 healthy children up to one year of age. We found that these MMPs and TIMPs are significantly (*P* ═ 0.000) higher in children with VSD, and the correlations between their concentrations and the size of the defect are positive, especially for MMP-9 and TIMP-1. MMP-9 was significantly (*P* ═ 0.044) higher in cases in which VSD did not close in the first year of life compared to cases in which it closed. Our results suggest the role of MMP-2, MMP-9, TIMP-1, and TIMP-2 in the aetiopathogenesis of VSD and that their urinary concentrations, especially of MMP-9, in combination with echocardiographic and clinical monitoring, could be useful in predicting its natural course.

## Introduction

Ventricular septal defect (VSD) is best defined as an abnormal congenital communication between the two lower chambers of the heart. With an estimated prevalence of about 3 per 1000 live births, it is, after the bicuspid aortic valve, the second most common congenital heart anomaly and is usually isolated [1–4]. VSD forms as a result of maldevelopment or an interruption in the formation of the interventricular heart septum within the first seven weeks of embryonic development. It mostly closes spontaneously within the first year; however, its etiology and pathogenesis are not fully understood [5, 6].

According to its location, VSD can be classified into four types. The perimembranous type is located in the posteroinferior quadrant of the septum and is primarily made up of fibrous tissue. The muscular type, whose borders are exclusively composed of muscular tissue, is located in the trabecular muscular part of the septum and can be further divided into apical, middle, anterior, and posterior sections. The inlet and outlet types, localized in the inlet and outlet septum of the right ventricle, respectively, are rare. Muscular and perimembranous VSD often close spontaneously during the first years of life in up to 82% of cases, often without the need for therapy [7–10]. In contrast, inlet and outlet defects usually always require surgical closure [8]. When surgical closure of the defect is necessary, if it is not performed in a timely manner, serious complications can occur, including Eisenmenger syndrome. For this reason, it is important to determine factors, i.e., biomarkers, that could help predict its natural course and ultimately assist in creating the best therapeutic approach.

Knowledge of the role of matrix metalloproteinases (MMPs) and tissue inhibitors of metalloproteinases (TIMPs) in extracellular matrix remodeling processes during embryogenesis was the reason to explore their influence on both physiological and pathological cardiogenesis. MMPs are a family of zinc-dependent endopeptidases that degrade various components of the extracellular matrix. The most studied MMPs are MMP-2 and MMP-9, whose elevated blood concentrations are strongly associated with heart remodeling disorders, which, for example, can lead to the dilatation of the left ventricle after myocardial infarction [10, 11]. Animal studies have found that MMP-2 plays a notable role in normal cardiogenesis [12–14]. Unlike the numerous studies on the role of MMPs in cardiovascular diseases, such as atherosclerosis, cardiac insufficiency, hypertension, aneurysm, and hypertrophic cardiomyopathy [15–20], studies examining their role in human cardiogenesis and congenital heart anomalies are rare. These studies reported that circulating levels of MMP-2 or MMP-9, or both, are associated with cardiac function and outcomes in isolated severe aortic stenosis, bicuspid aortic valve, tetralogy of Fallot, and other complex cyanotic heart anomalies [21–26]. As for studies on MMPs and TIMPs in isolated VSD, there are only a few reports in the literature, and in all of them, the levels were measured in blood samples [22–29].

More than 20 years ago, MMPs were detected in the urine of individuals suffering from malignant disease, which was expected due to the degree of tissue remodeling in these conditions [30]. However, the presence of MMPs in the urine of children with congenital anomalies was a surprising finding, first published by Marler with associates in 2005 [31]. These authors discovered that MMPs could be detected in the urine of patients born with vascular malformations, in their fully functional and intact forms, and that there was a positive correlation between their concentration and disease progression. However, to the best of our knowledge, no study has been published so far about the role of MMPs and TIMPs in VSD, nor in congenital heart anomalies in general, that measured their concentrations in urine.

The aim of this research was to investigate the role of MMP-2, MMP-9, TIMP-1, and TIMP-2 in the etiopathogenesis of VSD using urine samples as an innovative, non-invasive approach, and to determine if their concentrations in urine can be used as biomarkers for predicting the spontaneous course of this congenital heart defect.

## Materials and methods

### Study design and participants

This prospective study was performed at the Clinic for Children’s Diseases, University Clinical Centre of the Republic of Srpska, in Banjaluka, from 2017 to 2020. A total of 72 patients up to one year of age were included: 52 patients with isolated VSD (experimental group) and 20 healthy children (control group). In the experimental group, all patients had isolated VSD detected via Color Doppler echocardiography. Patients with defects that exceeded 66% of the aortic annulus (Ao) diameter (VSD/Ao > 0.66 mm) and those with inlet or outlet types of defects were not included from this study, as they usually require surgery rather than follow-up observation.

Since various factors, including infections, hypoxic-ischemic encephalopathy, and medications, may impact MMP and TIMP levels, this study exclusively enrolled patients who had an isolated VSD, no other congenital anomalies or diseases, and were not taking any medications, except for vitamin D in a dose to prevent rickets, at the time of taking the urine sample [32–34].

After obtaining written informed consent from parents, we collected clinical information, including each participant’s age (in days), gender, gestational age (in weeks), birth weight (in grams), and birth length (in centimeters). Body mass index (BMI) at birth was calculated using Quetelet’s formula, and nutrition status was determined according to gender-specific BMI-for-age percentiles in each case [35]. Participants were divided according to gender (male or female), gestational age (preterm: less than 37 weeks or term: from 37th to 42nd week), and nutritional status at birth (normal: BMI from the 10th to 90th percentiles or hypotrophic: BMI below the 10th percentile). In addition to that, participants from the experimental group were categorized by the size of the VSD (trivial, small, or large), its position in the heart septum (apical muscular, middle muscular, anterior muscular, or perimembranous) and the outcome at the end of the first year of life (VSD closed or VSD open). In our study, there were no participants who were born postterm, were hypertrophic at birth, or had a defect in the posterior muscular part of the ventricular septum. Participants from the control group were healthy children with normal echocardiographic findings. During their selection, we ensured that the control group did not differ from the experimental group by participant age, gender, gestational age, and nutritional status at birth. All participants with VSD and healthy controls were seen by a clinical geneticist, and in some cases, ultrasound examinations and cytogenetic tests were performed in order to exclude any additional anomalies.

### Echocardiography

VSD was detected by a cardiologist using conventional two-dimensional color Doppler echocardiography with a GE Healthcare - Vivid 4 ultrasound system and a 5-MHz linear sonde. The indication for echocardiography was a heart murmur noted during a regular physical examination. The defect type was defined according to its position in the septum. Its size was measured in all views, and the largest diameter, in millimeters (mm), was recorded. The aortic annulus (Ao) diameter was measured at the level of the valve and expressed in millimeters. Concurrently, additional cardiac anomalies were excluded. Echocardiography was also performed in all subjects from the control group to exclude any cardiac abnormalities. To negate the influence of weight and gestational age of the patient on the absolute VSD size, its size was determined in comparison to the Ao diameter (VSD/Ao ratio) [36]. VSD was considered trivial if it measured 25% or less of the Ao diameter (VSD/Ao ≤ 0.25 mm), small if it measured more than 25% but less or equal to 33% (0.25 mm **>** VSD/Ao ≤ 0.33 mm), and large if it measured more than 33% but less or equal to 66% of the Ao diameter (0.33 mm **<** VSD/Ao ≤ 0.66 mm). All patients from the experimental group were followed up periodically, every three to six months, until the end of the first year of life or until the defect’s closure. VSD was considered closed if the echocardiogram of the ventricular septum was normal, as confirmed by color Doppler flow mapping. Patients who required surgical closure of the defect were excluded from further study.

### Sample collection

For this study, we collected one-morning urine sample from each patient after VSD diagnosis, as soon as possible, and from all controls. Each sample, labeled with a number, was immediately stored in a 6 mL glass bottle in the freezer at a temperature of –18 ^∘^C to –20 ^∘^C until analysis.

### ELISA

Measurements of MMP-2, MMP-9, TIMP-1, and TIMP-2 in urine samples were performed at the Faculty of Medicine, University of Rijeka, using the ELISA method and high-selective kits produced specifically for that purpose (RayBio^®^ Human MMP-2 ELISA, Cat#: ELH-MMP2; RayBio^®^ Human MMP-9 ELISA, Cat#: ELH-MMP9; RayBio^®^ Human TIMP-1 ELISA, Cat#: ELH-TIMP1; RayBio^®^ Human TIMP-2 ELISA, Cat#: ELH-TIMP2) [37]. Urine samples were held at room temperature for 30 min for incubation. After preparing for analysis, standards (RayBio^®^) and urine samples were placed with micropipettes into the microplate (Microplate; RayBio^®^) and treated with specific human MMP-2, MMP-9, TIMP-1, and TIMP-2 antibodies. The proactive and active forms of enzymes from the samples were bound to antibodies on the plate walls. After wash-out, we added biotinylated enzymatic antibodies for MMP-2, MMP-9, TIMP-1, and TIMP-2 detection (Detection Antibodies MMP-2, MMP-9, TIMP-1, and TIMP-2; The RayBio^®^) and incubated for an hour at room temperature. Then, we washed out unbound antibodies from the plate, added 100 µL of streptavidin solution, an integral part of the ELISA kit, and incubated for 45 min. The plate was again washed, and TMB (tetramethyl-benzidine; RayBio^®^) was added, coloring the reaction between antibodies and enzymes in the sample blue. In the final phase, we added a Stop solution, which is also a part of the ELISA kit, that stops the reaction and changes its color from blue to yellow. Immediately after that, we read the intensity of the yellow color, which corresponds to the absorbance of the present and active forms of enzymes, on the Tecan sunrise absorbance microplate reader at a wavelength of 450 nm. For determining the mean absorbance value for each standard, sample, and blind probe, we used Magellan software.

### Ethical statement

The research was conducted in compliance with basic ethical and bioethical principles in accordance with the Nuremberg Code and the latest revision of the Declaration of Helsinki. All patients, i.e., parents or guardians, provided written informed consent. The study protocol was ethically reviewed and approved by the Ethics Committee of the University Clinical Centre of the Republic of Srpska (No: 01-9-396-2/17) and the Ethics Committee of the Faculty of Medicine, University of Banja Luka (No: 18/4.17/17).

### Statistical analysis

The obtained results were analyzed using Statistica 12 software (StatSoft, Inc., Tulsa, OK, USA). Indicators of descriptive statistics were used to display quantitative data. To compare differences between groups, we used chi-square and Fisher’s exact tests. Differences were calculated with the Mann–Whitney *U* test in the case of two small independent samples and with the Kruskal–Wallis ANOVA test in the case of multiple independent samples. Linear correlations were determined by the Pearson correlation coefficient. A *P* value of less than 0.05 was considered statistically significant.

## Results

### Clinical description of participants

The experimental group included 52 children with VSD, and the control group consisted of 20 healthy children, all aged less than one year. The groups did not differ in age, gender, gestational age, or nutritional status at birth (*P* ═ 0.47; *P* ═ 0.98; *P* ═ 0.36; *P* ═ 0.73, respectively). The clinical characteristics of both study groups are described in Table 1.

**Table 1:** Clinical characteristics of participants

	**Experimental group (*n* ═ 52)**	**Control group (*n* ═ 20)**	***P* value**
Age (days)	31.9 ± 59.9	34.6 ± 76.2	0.47
Gender (M/F)	27/25	11/9	0.98
Gestation age (term/preterm)	41/11	13/7	0.36
Nutrition status at birth (normal/hypotrophic)	41/11	15/5	0.73

Data are represented as absolute numbers or mean ± standard deviation. VSD: Ventricular septal defects; n: Number of participants; SD: Standard deviation; M: Male; F: Female.

Among participants from the experimental group, 21 (40.38%) had trivial VSD (VSD/Ao ≤ 0.25 mm), 12 (23.08%) had small VSD (0.25 mm < VSD/Ao ≤ 0.33 mm), and 19 (36.54%) had large VSD (0.33 mm < VSD/Ao ≤ 0.66 mm). There were no significant differences in age, gender, gestational age, or nutritional status at birth between groups with different sizes of VSD (*P* ═ 0.15; *P* ═ 0.56; *P* ═ 0.19; *P* ═ 0.61, respectively).

Apical muscular defect was present in 21 (40.38%) participants, middle muscular defect in 15 (28.85%), anterior muscular defect in 4 (7.69%), and perimembranous defect in 12 (23.08%) participants from the experimental group. The average size of VSD at the time of the diagnosis, expressed as a VSD/Ao ratio, was 0.32 mm ± 0.13 SD (range 0.57). The average size of the apical muscular defect was 0.26 mm ± 0.08 SD (range 0.3), middle muscle defect 0.31 mm ± 0.10 SD (range 0.22), anterior muscular defect 0.20 mm ± 0.06 SD (range 0.27), and perimembranous defect 0.49 mm ± 0.13 SD (range 0.44). The Kruskal–Wallis ANOVA test showed that there were significant size differences between apical muscular and middle muscular (*P* ═ 0.03), apical muscular and perimembranous (*P* ═ 0.00) and middle muscular and perimembranous (*P* ═ 0.00) defects.

### Levels of MMP-2, MMP-9, TIMP-1, and TIMP-2 in urine in the experimental and in the control group

**Figure 1.**
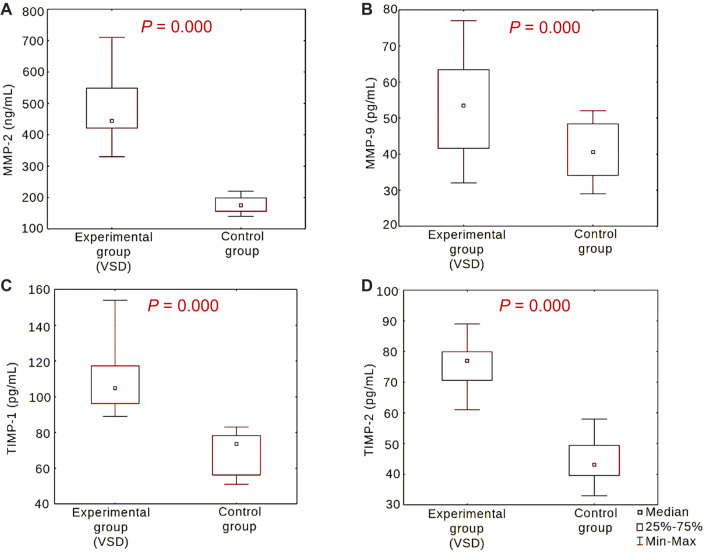
**Levels of MMP-2 (A), MMP-9 (B), TIMP-1 (C), and TIMP-2 (D) in urine in the experimental and control groups.** The Mann–Whitney *U* test was used for comparisons. VSD: Ventricular septal defect; MMP-2: Matrix metalloproteinase 2; MMP-9: Matrix metalloproteinase 9; TIMP-1: Tissue inhibitor of metalloproteinase 1; TIMP-2: Tissue inhibitor of metalloproteinase 2.

MMP-2, MMP-9, TIMP-1, and TIMP-2 levels in urine samples were significantly higher in the experimental group than in the control group (*P* ═ 0.000 for all) (Table 2 and Figure 1).

**Table 2:** Comparison of MMP-2, MMP-9, TIMP-1, and TIMP-2 levels in urine between the experimental group and the control group

	**Group**	***n***	**Rank sum**	***U***	***Z* value**	***P* value**
MMP-2	Experimental	52	2418.00	0.00	6.53	0.000*
	Control	20	210.00			
MMP-9	Experimental	52	2182.50	235.50	3.57	0.000*
	Control	20	445.50			
TIMP-1	Experimental	52	2418.00	0.00	6.53	0.000*
	Control	20	210.00			
TIMP-2	Experimental	52	2418.00	0.00	6.53	0.000*
	Control	20	210.00			

Data are represented as absolute numbers. Comparing is done with Mann–Whitney *U* test. Statistically significant differences are marked with an asterisk. MMP-2: Matrix metalloproteinase 2; MMP-9: Matrix metalloproteinase 9; TIMP-1: Tissue inhibitor of metalloproteinase 1; TIMP-2: Tissue inhibitor of metalloproteinase 2; n: Number of participants; *: *P* < 0.05.

### Levels of MMP-2, MMP-9, TIMP-1, and TIMP-2 in urine depending on the size of VSD

**Figure 2.**
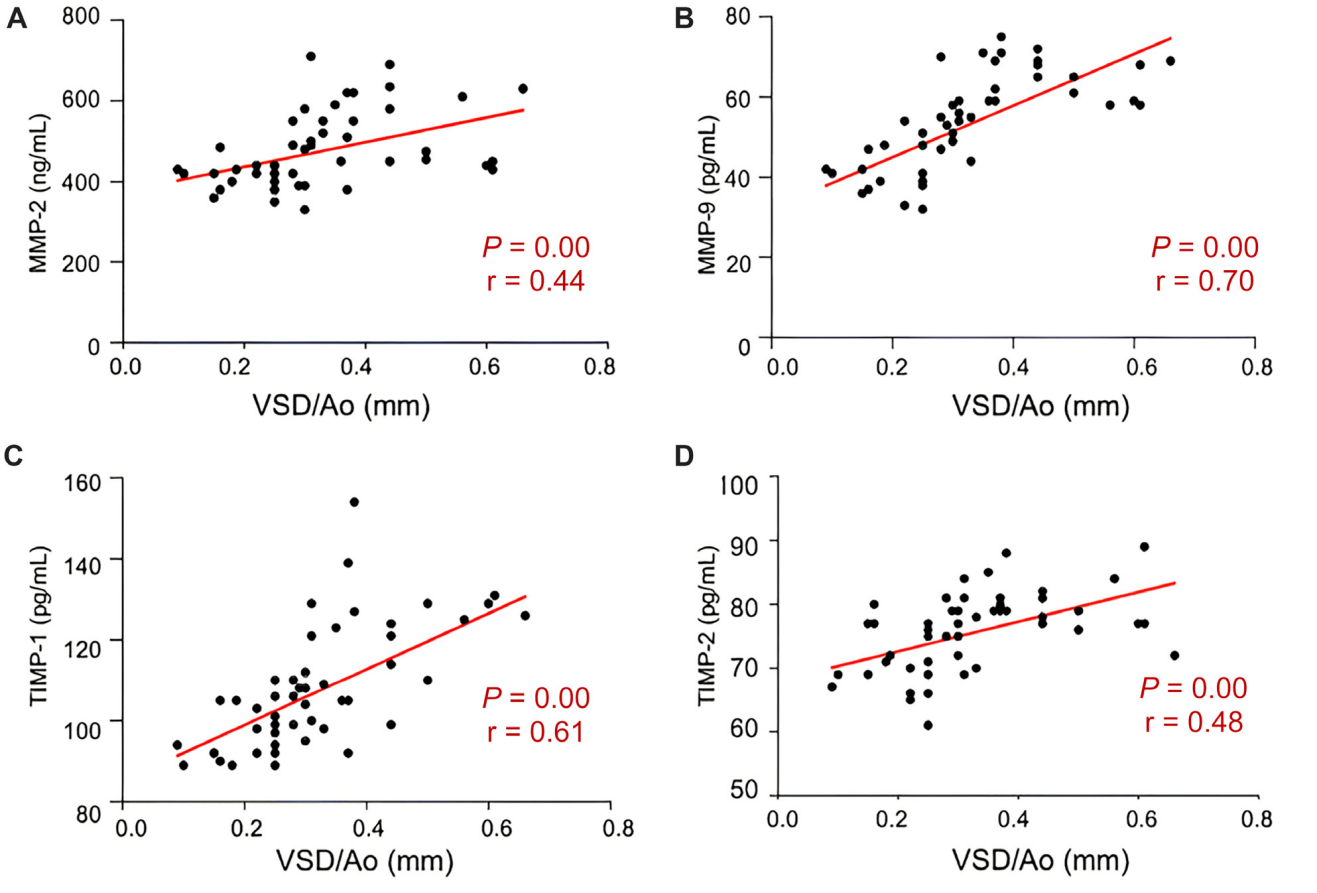
**Correlations between levels of MMP-2, MMP-9, TIMP-1, and TIMP-2 in urine and the size of VSD.** Linear correlations, determined by the Pearson correlation coefficient, between levels of MMP-2 (A), MMP-9 (B), TIMP-1 (C), and TIMP-2 (D) in urine and the size of VSD presented as VSD/Ao ratio. VSD: Ventricular septal defect; Ao: Aortic annulus; MMP-2: Matrix metalloproteinase 2; MMP-9: Matrix metalloproteinase 9; TIMP-1: Tissue inhibitor of metalloproteinase 1; TIMP-2: Tissue inhibitor of metalloproteinase 2; r: Correlation coefficient.

We found positive correlations between levels of MMP-2, MMP-9, TIMP-1, and TIMP-2 in urine and the size of VSD, with correlation coefficients of *r* ═ 0.44 for MMP-2 (Figure 2A), *r* ═ 0.70 for MMP-9 (Figure 2B), *r* ═ 0.61 for TIMP-1 (Figure 2C), and *r* ═ 0.48 for TIMP-2 (Figure 2D).

**Figure 3.**
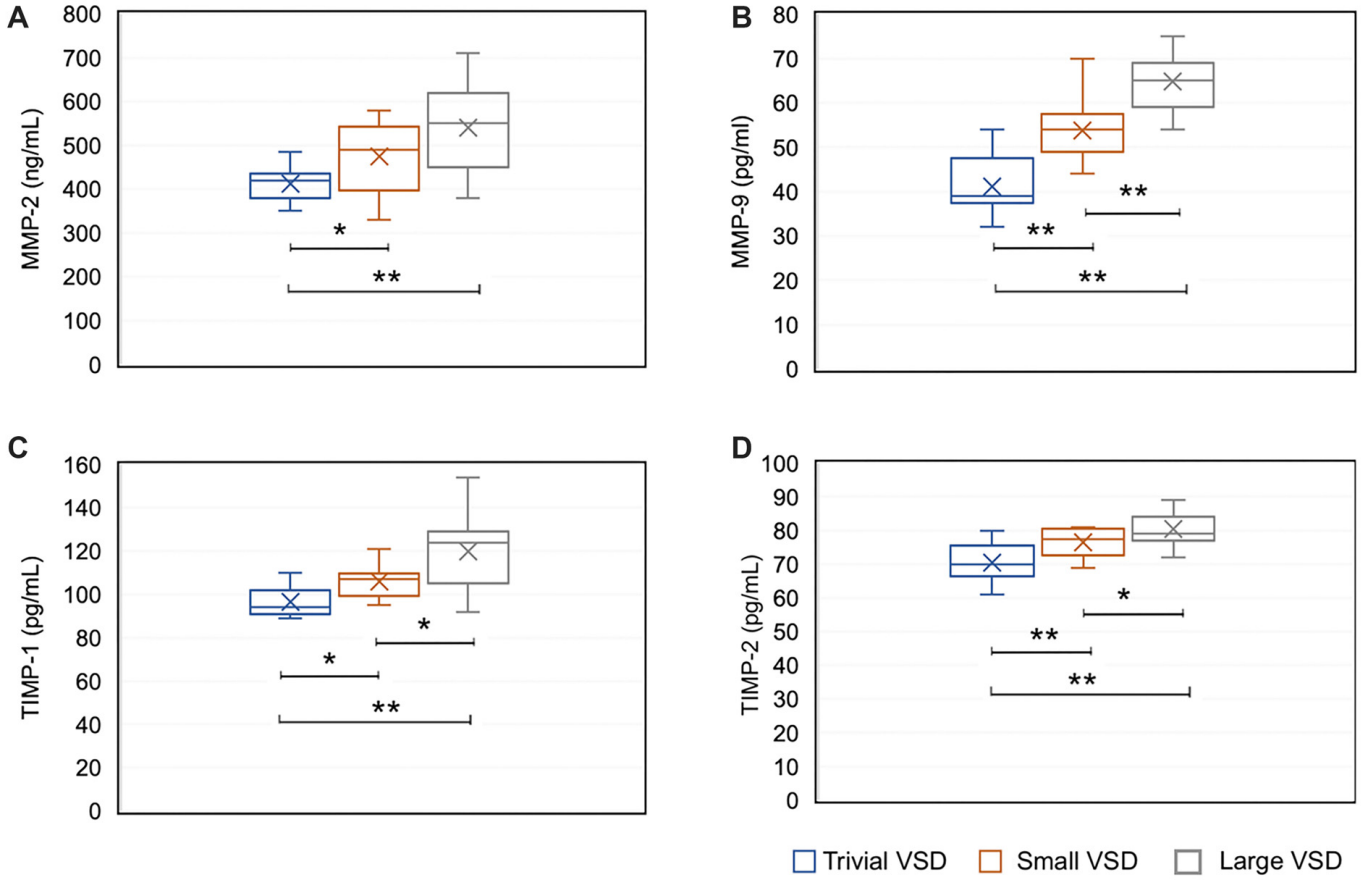
**Levels of MMP-2, MMP-9, TIMP-1, and TIMP-2 in urine based on the size of VSD.** Comparison of MMP-2 (A), MMP-9 (B), TIMP-1 (C), and TIMP-2 (D) levels in urine between groups with different VSD sizes was performed using the Kruskal–Wallis ANOVA test. Statistically significant differences are marked with an asterisk. VSD: Ventricular septal defect; MMP-2: Matrix metalloproteinase 2; MMP-9: Matrix metalloproteinase 9; TIMP-1: Tissue inhibitor of metalloproteinase 1; TIMP-2: Tissue inhibitor of metalloproteinase 2; *: *P* < 0.05, **: *P* < 0.01.

Urine levels of all studied MMPs and TIMPs were significantly different between groups with different sizes of the defect. MMP-2 levels were the lowest in patients with a trivial size of VSD and the highest in those with a large size of VSD (Figure 3A). Using the Kruskal–Wallis ANOVA test, we established that levels were significantly different between trivial and small VSD groups (*P* ═ 0.02) and between the trivial and large defect groups (*P* ═ 0.00), while the difference was not statistically significant (*P* ═ 0.10) between the small and large VSD groups. MMP-9 levels were lowest in patients with a trivial size of VSD and highest in those with a large VSD (Figure 3B). The difference in its levels was significant between all three groups (*P* ═ 0.00). Urine levels of both studied TIMPs were significantly different between all groups with different sizes of VSD (Figure 3C and 3D). The difference in TIMP-1 levels was significant between groups with trivial and small VSD (*P* ═ 0.02), small and large VSD (*P* ═ 0.00) and trivial and large VSD (*P* ═ 0.01). TIMP-2 urine levels were also significantly different among all groups. Between groups with trivial and small defects and groups with small and large defects, the difference was *P* ═ 0.00 and between groups with small and large VSD, the difference was *P* ═ 0.04.

### Levels of MMP-2, MMP-9, TIMP-1, and TIMP-2 in urine depending on the location of VSD

**Figure 4.**
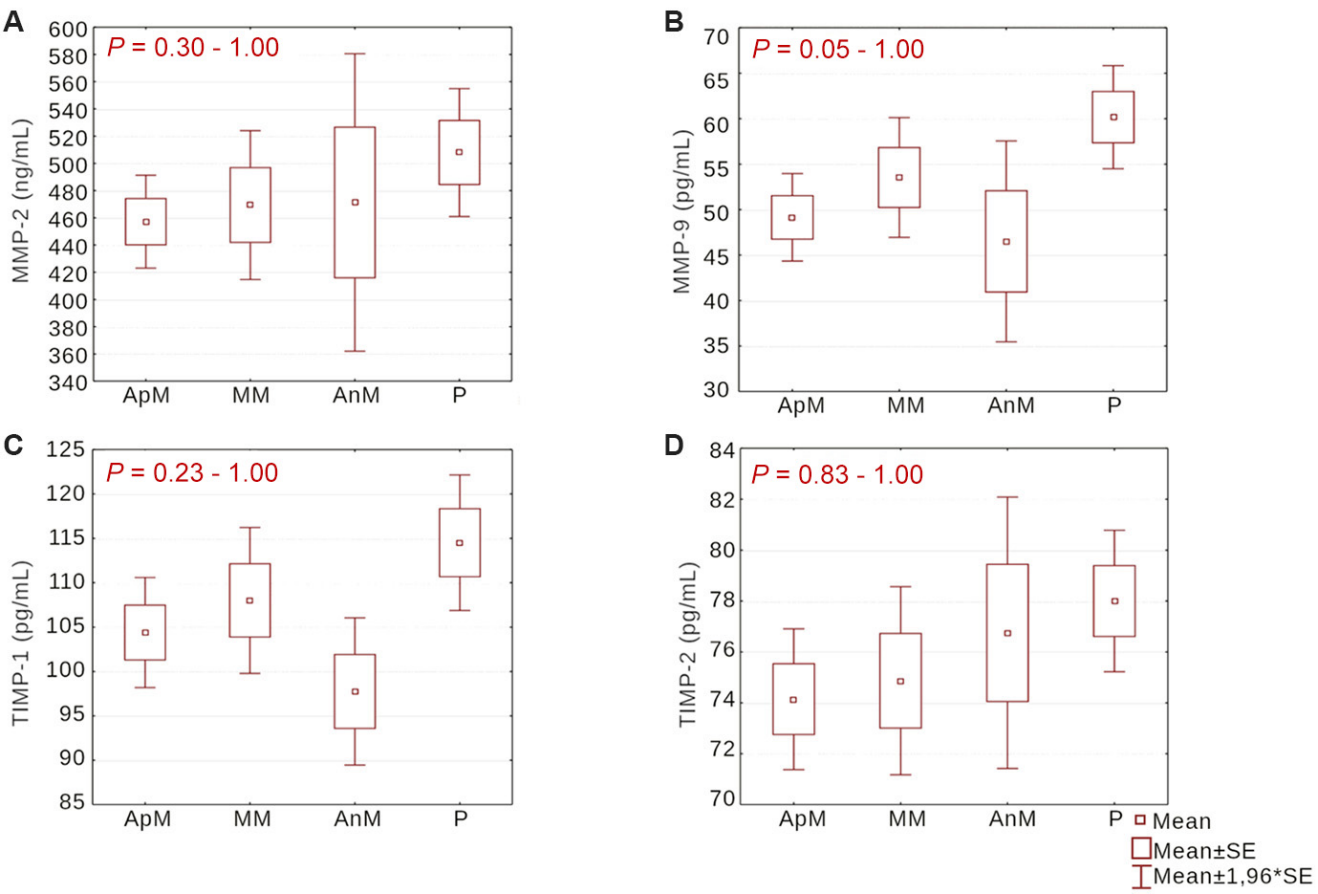
**Levels of MMP-2, MMP-9, TIMP-1, and TIMP-2 in urine based on the location of VSD.** Comparison of MMP-2 (A), MMP-9 (B), TIMP-1 (C), and TIMP-2 (D) levels in urine between groups with different VSD location was performed using the Kruskal–Wallis ANOVA test. MMP-2: Matrix metalloproteinase 2; MMP-9: Matrix metalloproteinase 9; TIMP-1: Tissue inhibitor of metalloproteinase 1; TIMP-2: Tissue inhibitor of metalloproteinase 2; ApM: Apical muscular; MM: Middle muscular; AnM: Anterior muscular; P: Perimembranous.

Using the Kruskal–Wallis ANOVA test, we found that levels of the studied MMPs and TIMPs in urine did not differ significantly between groups with different locations of VSD, i.e., between the apical muscular, middle muscular, anterior muscular and perimembranous VSD groups. The differences ranged from *P* ═ 0.30 to *P* ═ 1.00 in the case of MMP-2 (Figure 4A), from *P* ═ 0.05 to *P* ═ 1.00 for MMP-9 (Figure 4B), from *P* ═ 0.23 to *P* ═ 1.00 for TIMP-1 (Figure 4C) and from *P* ═ 0.83 to *P* ═ 1.00 for TIMP-2 (Figure 4D).

### Levels of MMP-2, MMP-9, TIMP-1, and TIMP-2 in urine and spontaneous closure of VSD

In 32 (61.54%) participants from the experimental group, VSD spontaneously closed within the first year of life, while in 20 (38.46%), it did not. Closure rates were significantly different in groups with different defect sizes (*P* ═ 0.002): trivial defects closed in 85.71% of cases, small in 66.67%, and large in 31.58% of cases.

**Figure 5.**
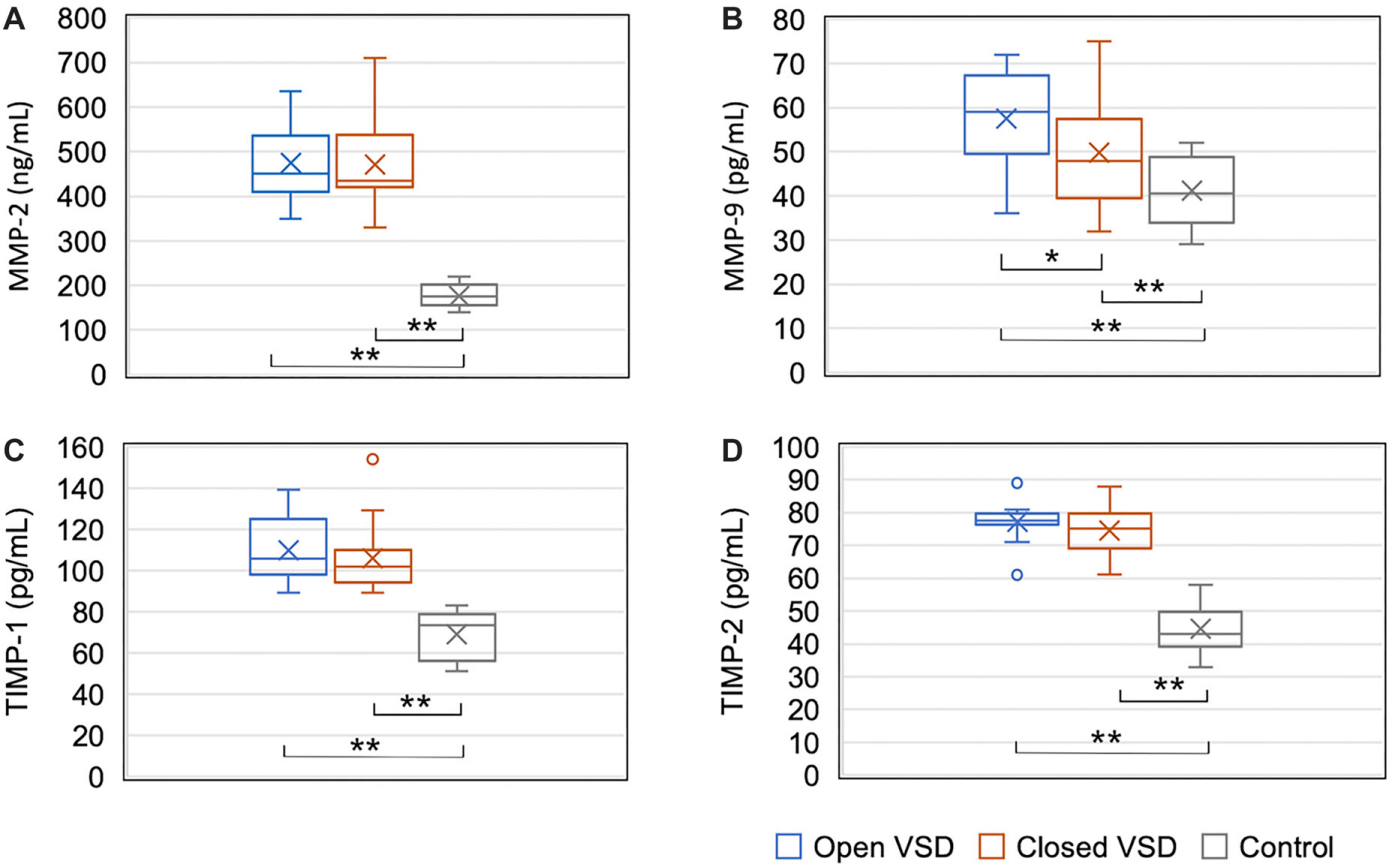
**Levels of MMP-2, MMP-9, TIMP-1, and TIMP-2 in urine based on the outcome of VSD at the end of the first year of life.** Comparison of MMP-2, MMP-9, TIMP-1, and TIMP-2 levels between the group of patients with VSD that did not close, the group of patients with VSD that spontaneously closed, and the control group was performed using the Kruskal–Wallis ANOVA test. Statistically significant differences are marked with an asterisk. MMP-2: Matrix metalloproteinase 2; MMP-9: Matrix metalloproteinase 9; TIMP-1: Tissue inhibitor of metalloproteinase 1; TIMP-2: Tissue inhibitor of metalloproteinase 2; *: *P* < 0.05; **: *P* < 0.01.

Urine levels of MMPs and TIMPs were compared between the group with VSD that did not close and the group with VSD that closed spontaneously within the first year of life (Table 3). Levels of MMP-9 were significantly higher (*P* ═ 0.044) in the group of participants whose VSD did not close, while levels of MMP-2, TIMP-1, and TIMP-2 were not significantly different between these two groups (*P* ═ 0.337; *P* ═ 0.318; *P* ═ 0.112, respectively) (Table 2). Both groups showed significant differences (*P* ═ 0.000) from the control group in levels of MMP-2, MMP-9, TIMP-1, and TIMP-2 (Figure 5).

**Table 3:** Comparison of urine levels of MMP-2, MMP-9, TIMP-1, and TIMP-2 between the group with VSD that did not close and the group with VSD that did spontaneously close

	**Group**	***n***	**Rank sum**	***U***	**Z value**	***P* value**
MMP-2	Open VSD	20	581.50	268.50	0.96	0.337
	Closed VSD	32	796.50			
MMP-9	Open VSD	20	637.50	212.50	2.01	0.044*
	Closed VSD	32	740.50			
TIMP-1	Open VSD	20	583.50	266.50	0.99	0.318
	Closed VSD	32	794.50			
TIMP-2	Open VSD	20	615.00	235.00	1.59	0.112
	Closed VSD	32	763.00			

Data are represented as absolute numbers. Comparing is done with the Mann–Whitney *U* test. Statistically significant difference is marked with an asterisk. MMP-2: Matrix metalloproteinase 2; MMP-9: Matrix metalloproteinase 9; TIMP-1: Tissue inhibitor of metalloproteinase 1; TIMP-2: Tissue inhibitor of metalloproteinase 2; VSD: Ventricular septal defects; n: Number of participants; *: *P* < 0.05.

## Discussion

In this study, we investigated the role of MMP-2, MMP-9, TIMP-1, and TIMP-2 in the etiopathogenesis of VSD and their potential as biomarkers for disease outcomes, using urine samples. Since no previous research on this topic has analyzed their concentrations in urine, we compared our results with studies about congenital heart defects, especially VSD, where MMPs and TIMPs were measured in blood, as well as with studies analyzing urinary MMPs and TIMPs in other diseases.

We found that MMP-2, MMP-9, TIMP-1, and TIMP-2 levels in urine were significantly higher (*P* ═ 0.000) in children with isolated VSD compared to healthy children, and that the correlation between these levels and the size of VSD is a positive. The correlation was higher in cases for MMP-9 and TIMP-1 than in cases for MMP-2 and TIMP-2. Cheng et al. [28] also found that MMP-2 and MMP-9 in the blood of children with VSD are higher than in healthy children, with MMP-9 levels positively correlated with VSD size. On the other hand, the same authors did not find a correlation between the concentration of MMP-2 in blood and the size of the defect [28]. Kiliç et al. [22] found higher blood concentrations of these MMPs, as well as TIMP-1, in children born with heart anomalies compared to their concentrations in healthy children, but only MMP-2 levels were significantly different. In the same study, the authors also found positive correlations between MMP-2 and TIMP-1 levels and echocardiographic parameters. For TIMP-2, they did not find that its concentrations to differ from those in healthy children. Cheung et al. [25] studied MMP-2 and MMP-9 levels in blood samples from patients with surgically corrected cardiac anomalies, such as tetralogy of Fallot, and found significant increases compared to healthy individuals, leading us to the conclusion that surgical correction of the anomaly does not affect their level.

The results of our study and those of related studies indicate that MMP-2, MMP-9, TIMP-1, and according to our findings, also TIMP-2, play a role in the etiopathogenesis of VSD and other congenital heart anomalies in humans. Their high concentrations indicate increased extracellular matrix degradation affecting myocardial remodeling, which may lead to myocardial structural abnormalities. The explanation of the connection between increased degradation of the components of the extracellular matrix and VSD may be explained by volume and pressure loading of the right ventricle, which in this congenital heart defect can result from the existence of a left–right shunt. It is known that this overload is accompanied by ventricular myocardium remodeling, but the mechanisms contributing to this progressive process are not fully understood. Spinale with associates reported that in ventricular myocardial remodeling caused by chronic volume overload, partial extracellular matrix degradation is primarily associated with increased MMP activity [38]. However, it is believed that many types of MMPs have specific roles in myocardial remodeling and that different factors selectively affect their activity and expression [39]. Nagatomo et al., in experiments on dogs, found that MMP-9 activity increases more than threefold during acute volume overload of heart chambers, but then returns to normal levels. They also found that MMP-1 activity decreases by 50% with volume increase in the left ventricle but not with pressure increase, as well as that MMP-3 activity increases more in chronic than in acute pressure elevation in heart chambers [40].

MMPs contribute to the pathophysiology of VSD through their roles in inflammation, cellular behavior, and vascular changes. They can modulate the inflammatory response by processing pro-inflammatory cytokines and chemokines, which can exacerbate cardiac tissue damage. By influencing the migration and proliferation of cardiac fibroblasts, MMPs are involved in repair mechanisms in the heart, such as VSD closure, but they can also lead to pathological remodeling, heart failure, or other complications associated with VSD. MMPs also play a role in remodeling the vascular structures within the heart and pulmonary circulation, that can result in pulmonary hypertension, a complication in patients with large VSDs.

MMP and TIMP knockout mice are often used to study various biological processes, including cardiac conditions, such as chronic pressure overload and myocardial infarction [41, 42]. However, neither of these studies reports VSD or other heart defects in these knockouts in a normal, healthy state. There are a few possible explanations for this. Deficiency of these MMPs or TIMPs might be compensated by other proteases, maintaining normal heart structure, or the timing of MMP expression may differ from critical periods for cardiogenesis in mice. However, under certain conditions, like myocardial infarction or heart failure, MMP-9 knockout mice can show differences in cardiac remodeling and function. This suggests that while MMP-9 may not be essential for normal heart development, but it does play a role in response to cardiac stress [42]. On the other hand, it seems that the role of genes, such as *GATA4, TBX5, NKX2-5, HOMEZ, PLAGL1,* and *CITED2,* in heart development is significant, as numerous studies on knockout mice report that mutations in these genes are frequently associated with VSD or other congenital heart anomalies [43–45].

We analyzed whether MMP and TIMP concentrations in urine relate to the location of defects in the interventricular septum. Although the difference is not statistically significant, we found higher levels of MMP-2, MMP-9, TIMP-1, and TIMP-2 in urine from patients with a defect in the perimembranous part of the septum compared to those with defects in the muscular parts of the septum. A possible explanation for this result is the statistically greater average size of the defect in the perimembranous part of the septum in our study, as we already found a positive correlation between urinary concentrations of these MMPs and TIMPs and the size of the defect. Another reason could be differences in remodeling processes of the extracellular matrix, which might be explained by histologically distinct borders of perimembranous and muscular defects. Baggen et al. [46], in a study on adult patients with congenital heart anomalies, also did not find that circulating levels of MMP-2, MMP-9, and TIMP-1 depend on this echocardiographic parameter. Among the 52 children with VSD, the defect spontaneously closed within the first year of life in 32 (61.54%) cases. This result is similar to the results of other studies conducted during the same period. For example, Cresti et al. [9], in a study published in 2018, found that the spontaneous closure rate of isolated VSDs in the first year is 60.2%.

In order to explore if these MMPs and TIMPs can be used as predictors of the spontaneous course of VSD, we compared their concentrations in the urine of children whose defects spontaneously closed compared to children in whom it persisted during the first year. We found that levels of MMP-9 in urine samples taken at the time of diagnosis were significantly higher in children where the VSD did not close compared to its levels in children where the defect did close. These children without proper treatment could eventually develop heart failure, so we assume that urinary MMP-9 levels could indicate disease outcome. This finding aligns with research that measured levels of MMP-9 in the blood of children with rheumatic heart disease and found elevated levels in those who developed congestive heart failure [47]. Another study, which explored the potential significance of MMP and TIMP levels in the blood of adult patients with heart failure, found that MMP-9, along with MMP-2 and TIMP-1, serves as a mortality predictor [19]. High circulating levels of MMP-9 have also been linked to increased mortality in patients with coronary artery disease [48]. In our study, MMP-2, TIMP-1, and TIMP-2 levels in urine did not significantly differ between children in whom the defect closed and those in whom it stayed open. Cheng et al. [28] also reported that the MMP-2 levels in the blood did not correlate with defect closure rates.

The role of MMPs in the spontaneous closure of VSD may be explained by their involvement in the mechanism of wound healing, specifically during the extracellular matrix remodeling phase [49, 50]. MMPs are essential for breaking down various components of the extracellular matrix, facilitating tissue remodeling, which is crucial for heart healing processes. They also regulate signaling pathways that affect cellular proliferation and apoptosis, modulate inflammatory responses, and support new tissue formation. MMPs also activate growth factors (such as TGF-β), further stimulating extracellular matrix production and tissue repair processes. Many MMPs, including MMP-9, increase during the third or maturation/remodeling phase of wound healing. Thus, targeting MMP activity with MMP inhibitors, like batimastat (BB-94), has been found to delay the wound healing process and can be used as targeted therapy for many different types of diseases, including cancers, idiopathic pulmonary fibrosis, and diabetic wounds [51, 52]. ND-336, another MMP inhibitor selective for MMP-2, -9, and -14, was found to accelerate diabetic wound healing in a study on animals [53]. According to the study, ND-336 reduces inflammation and intensifies angiogenesis and re-epithelialization of the wound [53].

Numerous studies indicate that, alongside MMP and TIMP, several other inflammatory markers, including N-terminal pro-B-type natriuretic peptide (NT-proBNP), high-sensitive C-reactive protein (hsCRP), soluble suppression of tumorigenicity-2 (sST2), and high-sensitive troponin T (hs-TnT), have independent prognostic value in patients with congenital heart diseases [54–60]. In patients with VSD, Zhang et al. [61] found an alteration in proteins that, as acute-phase reactants to various forms of tissue injury, trigger immune responses. Including other biomarkers in the study of VSD could give better insights into the inflammatory activity in this congenital heart defect, following examples of studies that investigated multiple inflammatory biomarkers in other congenital heart anomalies [62, 63].

Our study acknowledges several limitations. First, numerous physiological processes in the first months of life may affect both MMP and TIMP concentrations. Second, the absence of previous research on this topic, a relatively small sample size, and groups not well-matched in participant numbers posed challenges for interpreting results. Finally, we measured MMP and TIMP levels in only one urine sample per patient. Multiple measurements during the monitoring period would allow a better understanding of the correlation between their concentrations in urine and the natural course of VSD. The main reason we could not include more participants in the study and collect more urine samples during follow-up was the limited number of ELISA kits that we had. All these limitations collectively highlight areas for future study and discussion of our findings.

## Conclusion

Our limited study suggests that MMP-2, MMP-9, TIMP-1, and TIMP-2 play roles in the etiopathogenesis of VSD, particularly MMP-9 and TIMP-1. We also conclude that urinary concentration of MMP-9, along with echocardiographic and clinical findings, may be helpful for predicting the natural course of VSD and, thus, assist in creating the best follow-up and therapy plan for each patient with this congenital heart defect.
